# Self-care in haemodialysis treatment tasks in community and hospital-based units: A cross-sectional study

**DOI:** 10.1371/journal.pone.0325940

**Published:** 2025-06-10

**Authors:** Chava Kurtz, Efrat Shadmi, Karl Skorecki, Etty Kruzel-Davila, Alon Antebi, Tatyana Tsehovsky, Sivan Spitzer

**Affiliations:** 1 Haemodialysis Unit, Galilee Medical Center, Nahariya, Israel; 2 Azrieli Faculty of Medicine, Bar Ilan University, Sefad, Israel; 3 The Cheryl Spencer Department of Nursing, Faculty of Social Welfare and Health Sciences, University of Haifa, Haifa, Israel; 4 Rappaport Faculty of Medicine, Technion – Israel Institute of Technology, Haifa, Israel; 5 Rambam Health Care Campus, Haifa, Israel; 6 Fresenius Medical Care, Pardes-Hana, Israel; 7 Haemodialysis Unit, Edith Wolfson Medical Center, Holon, Israel.; Warren Alpert Medical School of Brown University: Brown University Warren Alpert Medical School, UNITED STATES OF AMERICA

## Abstract

**Background:**

Patient self-care improves outcomes in chronic diseases, yet patients in haemodialysis units tend to be passive recipients of treatments.

**Purpose:**

To explore patient self-care in haemodialysis tasks and identify factors influencing interest and participation.

**Methods:**

Patient interest and participation were assessed using a Likert-type scale. Associations were tested with bi-variate analysis and logistic regression, accounting for hemodialysis unit type.

**Results:**

Questionnaires from 339 patients in hospital and community-based units showed 89.1% expressed interest in care tasks. Lower education (OR 0.36, 95% CI 0.15–0.9) and being single (OR 0.27, 95% CI 0.12–0.6) decreased interest. Participation in treatment tasks was observed in 40.1% of patients. Lesser odds of participation were seen amongst those of Jewish religion (OR 0.3, 95% CI 0.02–0.54), and dialyzing in community units (OR 0.44, 95% CI 0.25–0.76), whereas higher odds were seen in people reporting higher economic status (OR 2.67, 95% CI 1.44–4.93), venous access via arteriovenous shunts (OR 3.31, 95% CI 1.98–5.54), more years on dialysis (OR 1.86, 95% CI 1.06–3.24), and participants expressing interest in participation (OR 6.01, 95% CI 2.18–16.53). A larger proportion of patients who were not interested in participation were from community-based units (75.7%), compared to those who expressed at least some interest (61%).

**Conclusions:**

While most patients expressed interest in participating, only a minority actually participated. There is need for greater engagement of interested patients. Organizational factors play an important role in determining actual participation, above and beyond personal patient factors. Patients should be presented with the opportunity to participate according to their interest and capabilities.

## Introduction

Chronic comorbidities, such as diabetes and hypertension, are common in patients with chronic kidney disease. In managing these conditions, patients often engage in self-care at home, performing various degrees of maintenance, monitoring, and management of complex treatment protocols [1]. However, a paradox emerges when these same individuals transition to in-center dialysis units—where they frequently adopt a passive role, refraining from active participation in even the most basic tasks, such as measuring weight or blood pressure—tasks that they routinely perform independently for their other chronic conditions [2,3]. This contrast in behavior raises important questions about patient participation in dialysis settings. Therefore, this study aims to explore patient participation within in-center hemodialysis units and examine the various factors that may contribute to its absence or presence.

The prevalence of patients with Stage V Chronic Kidney Disease (End Stage Kidney Disease) requiring renal replacement therapy is increasing worldwide [4,5]. Globally, about 90% of patients on dialysis receive hemodialysis, most commonly in-center dialysis delivered in hospital or community-based units several times a week for 3-to-5-hour sessions. This routine places a significant burden on patients, caregivers, and healthcare systems [5,6]. Hemodialysis treatment involves a range of tasks, some relatively simple and others requiring specialized clinical knowledge. These include basic tasks that are commonly performed by individuals with multi morbidity and chronic illnesses as part of daily care routines, such as weighing, measuring and recording vital signs (termed “simple tasks” heretofore). More complex tasks require specific technical and clinical knowledge, such as preparing the dialysis machine and equipment and attaining vascular access through an arteriovenous shunt or a central venous catheter (CVC) (termed “complex tasks” heretofore). Despite the ability of patients to manage similar health-related tasks independently at home, in the context of in-center dialysis care, the vast majority of these tasks are performed exclusively by healthcare providers—most commonly nurses or technicians [7]. Consequently, patient participation in their own care is not typically encouraged, even for tasks that they are already familiar with in other aspects of their health management. For example, simple tasks such as self- weighing and blood pressure monitoring -- routinely performed by patients with congestive heart failure [8] or hypertension [9] as part of their ongoing self-care – are often discouraged or even prohibited when they enter the dialysis unit. Even more complex tasks, such as inserting drugs/fluids into a CVC as part of home Total Parenteral Nutrition (TPN) treatment are becoming increasingly more common in home-care settings [10], yet patients are not typically allowed to manage their CVCs once they are in a dialysis unit. Commonly, the dominant culture in many dialysis units remains one of dependency and passivity [11,12].

Although patients in dialysis units often receive care in a passive manner, there is clear evidence that they are capable of actively participating in treatment-related care tasks, particularly in home-dialysis care. Studies have shown that patients managing their own dialysis at home successfully perform even complex clinical and technical tasks, such as inserting needles into a fistula, connecting a central catheter to the dialysis machine, and administering medications independently [11]. Despite this demonstrated capability, such self-management practices are often not encouraged in in-center dialysis settings, where patients generally take on a more passive role. Research further suggests that structured self-management support programs can significantly enhance patients’ ability to engage in their care, leading to improvements in self-care behaviors and clinical outcomes, particularly for individuals managing multiple chronic conditions such as diabetes and chronic kidney disease [12].

Research also indicates that patient engagement in self-management is associated with better health outcomes and quality of life, reinforcing the potential benefits of fostering greater participation even within in-center dialysis units [12]. Programs which incorporate education, goal-setting, and active patient involvement have shown improvements in adherence and clinical indicators, such as blood pressure and metabolic control. These findings reinforce the potential benefits of promoting patient participation, even in in-center dialysis units where opportunities for engagement may be more limited.

Acknowledging the benefits of patient participation in their care, models of “shared hemodialysis care” have been developed. This describes a modality of self-management under supervision, where patients are given the opportunity to learn and perform tasks related to their hemodialysis treatment [14,15]. This approach is centered on patient empowerment, allowing individuals to take on tasks and responsibilities aligned with their comfort and capabilities, in an environment designed to foster active participation [13,14]. Shared hemodialysis care has been associated with improvements in both patient and staff satisfaction, as it shifts the focus away from purely technical aspects of care and allows nursing staff to adopt a more educational and supportive role [14,15]. Studies have found that patients involved in shared hemodialysis care demonstrate better clinical outcomes, including higher serum albumin levels, lower serum phosphate, and reduced need for antihypertensive medications when compared with patients of similar age and frailty to patients who do not participate in shared care [16]. Beyond these individual benefits, shared care models may also contribute to enhanced patient safety, equity, and efficiency within dialysis units by fostering a deeper understanding of treatment among patients and tailoring care to meet individual needs and abilities [17]. Despite these advantages, widespread adoption of shared care practices remains limited, with patient participation in dialysis-related tasks within in-center settings continuing to be uncommon [18,19].

With the growing realization of the benefits of patient participation and self-management in dialysis care, it has become increasingly evident that participation rates vary widely among patients. Factors found to influence patients’ ability and motivation to self-manage include demographic variables, such as socioeconomic status and health literacy, as well as clinical factors like multi morbidities and treatment complexity [18,20]. A large study found that patients who engaged more frequently in shared hemodialysis care were typically younger, less frail, had fewer co-morbidities, and demonstrated greater physical strength [16]. Additionally, differences in organizational characteristics have been observed, particularly between hospital-based and community-based dialysis units. Patients in community-based units in general tend to be younger and healthier overall [21]. While these differences underscore the role of patient characteristics in participation, current research has primarily focused on individual factors rather than examining the broader impact of organizational culture and policies. To address this gap, this study aims to gain insight into patient participation in hemodialysis treatment-related tasks within both hospital-based and community-based settings. Specifically, we assess what patient-related and unit related factors are associated with interest in and/or actual participation in hemodialysis treatment care tasks. We hypothesize that younger patients, those with fewer comorbid conditions, those with higher health literacy, and those receiving care in community-based units will demonstrate higher levels of interest in participation and higher actual participation rates.

## Methods

### Study design

This was a multi-center, cross-sectional, questionnaire-based study with a descriptive exploratory design.

### Setting and participants

All eligible patients from four hemodialysis units in Israel, treated between May 2022 and May 2023, were invited to participate in the study. Recruitment took place from May 1, 2022 to May 31, 2023. Patients were eligible if they were over the age of 18, capable of communicating in one of the study’s included languages (Russian, Hebrew, or Arabic), and were established on hemodialysis treatments. Patients were excluded if they had cognitive disabilities, were acutely ill during the study period, or had previously participated in similar research, to avoid potential burden to patients.

The study included four hemodialysis units: two government-owned, hospital-based units, and two community-based units owned by a private global company. Care in both types of units is part of the basic coverage provided under Israel’s National Health Insurance Law, through membership in one of four non-profit health funds that serve as insurers and providers of services. Health funds establish contracts with both private and public organizations, but these arrangements are generally unknown and inconsequential to patients at the point of care. All units have a predetermined nurse-to-patient ratio of approximately 2:8, and nurses hold advanced acccreditation in nephrology. Home hemodialysis is not commonly available in Israel, and none of the four units involved in this study offer home hemodialysis.

The minimum required sample size was determined using an a priori G*power calculation (version 3.1.9), with an alpha error probability of <0.05 and power >0.80, yielding a requirement of 208 participants.

### Data collection

Eligible study participants were recruited during hemodialysis treatments in the four participating hemodialysis units by a nurse in the unit. Upon obtaining patient consent, a research assistant – who was not previously known to the patients – conducted one-on-one administration of a digital questionnaire. Data collection took place between May 2022–2023. Questionnaires were composed of three sections: (1) Patients’ actual participation in hemodialysis treatment tasks over the previous three treatments; (2) Patient interest in participating in treatment-related tasks; (3) Sociodemographic and clinical information.

Interest in participation was measured using a slightly modified version of the Hemodialysis-Self-Care Activity List Evaluation (HD-SCALE) [22], a validated tool for assessing patients’ interest and perceived capability in performing hemodialysis-related care tasks. The HD-SCALE consists of ten treatment tasks, with each task rated on a Likert-type scale to indicate the level of interest and perceived capability (where 1 = not interested/no perceived capability, and 5 = already performing the task independently.) Previous research demonstrated good reliability for the HD-SCALE, with a Cronbach’s alpha range of 0.88–0.91.

For this study, an additional task - “responding to machine alarms” – was included based on clinical practice observations and consultation with nephrology nurses. Furthermore, to enhance clarity and remove a neutral midpoint, the Likert scale was modified to range from 1 (not interested in participating) to 4 (performing the task independently). The modified HD-SCALE demonstrated high internal reliability in this study, with a Cronbach’s alpha of 0.95. To analyze interest in participation, responses were dichotomized into two categories: “not interested” and “any level of interest”. This approach was chosen because individuals showing even slight or moderate interest may represent potential participants.

A separate checklist was used to assess actual participation in care tasks over the previous three treatments. This checklist was originally based on HD-SCALE items and piloted with several patients in one unit. The pilot phase revealed gaps in capturing certain aspects of treatment participation, leading to its expansion to include additional elements – most notably, decision-making tasks (e.g., adjusting ultrafiltration volume and treatment duration) and preparation tasks (e.g., washing the arteriovenous shunt site or changing CVC dressings). Content validity was established through an independent review by three experienced nephrology nurses, who refined the checklist until consensus was reached on its comprehensiveness. Following this process, 14 items were retained.

Participants rated each task on a 4-point Likert scale (1 = never to 4 = always). To evaluate any level of participation, responses were dichotomized into two categories: “never participate” and “any degree of participation”. Participation was defined as engagement in at least two care tasks, following a similar approach to Fotheringham et al. [11]. This threshold was set to ensure that participation extended beyond a single simple task, such as measuring weight. Since many patients may report “measuring weight” while passively standing on the scale without actively engaging in the process, the requirement of at least tasks provided an more reliable indicator of participation.

To validate this approach, a random sample of 20 patients was selected, and their dialysis nurses were asked to confirm whether the patients engaged in at least some aspects of their hemodialysis care. The nurses’ assessments fully matched the patients’ self-reports.

Health literacy was measured using Chew’s health literacy screening questions, (1. “How often do you have problems learning about your medical condition because of difficulty understanding written information?” 2. “How often do you have someone help you read hospital materials?” 3. “How confident are you filling out medical forms by yourself?”) [23]. The Cronbach’s alpha for this tool in this study was 0.9, reflecting high reliability. Sociodemographic and clinical information collected included age, years of education, marital status, vascular access type, and duration of hemodialysis treatment.

### Data analysis

Patient characteristics and outcomes for interest in participation and actual participation are presented as means ± standard deviations (for continuous variables) and counts/percentages (for categorical variables). Missing data accounted for less than 3% of responses and were handled using simple imputation, where missing values were replaced with the mean for continuous variables or the mode for categorical variables.

Bivariate analyses were conducted to examine associations between interest in participation, actual participation, and sociodemographic/clinical characteristics. A binomial logistic regression model was used to assess whether demographic and clinical characteristics predicted interest and actual participation in care tasks. Only variables with a significance level of p < 0.1 in bivariate analysis were included in the logistic regression model. The results are reported as odds ratios (OR) with 95% confidence intervals (CI). All analyses were conducted using IBM SPSS Statistics (version 29, IBM Corporation).

### Ethical considerations

The study received ethical approval from the Helsinki Committees of the participating hospitals (0196-21-NHR; 0142-22-WOMC) and from the University Faculty of Medicine’s Ethical Review Board for the participating community units (IRB 6–2022). Participants did not provide signed informed consent as the questionnaires were completed anonymously, and an exemption was received from the ethical review boards because of this. Participants were informed that their participation was voluntary.

## Results

### Sample characteristics

Of the 533 patients screened for participation in the study, 339 (63.6%) completed the questionnaire. The average age of the patients was 66.05 years (SD = 13.88), and approximately 44% had completed a high school education level. A detailed breakdown of participant demographics is provided in Table 1.

**Table 1:** Participants demographic and clinical characteristics.

Patient characteristics (*N* = 339)	*N*	*%* ^*^
Demographics		
Age, years, mean (SD)	66.05	13.97
Education level		
Elementary school or less	101	*29.8*
High school	149	*44*
Higher education	89	*26.3*
Sex		
Male	184	*54.3*
Female	154	*45.4*
Religion		
Jewish	151	*44.5*
Muslim	124	*36.6*
Other	63	*18.5*
Marital status		
Married/ in partnership	223	*65.8*
Single/ Divorced/ Widowed	115	*33.9*
Economic status		
Below average	42	*12.4*
Average	225	66.4
Above average	72	*21.2*
Health literacy (mean, SD)	3.01	1.59
Low	158	*46.6*
Average	37	*10.9*
High	144	*42.5*
Clinical characteristics		
Vascular access (CVC vs shunt)		
CVC	141	*41.6*
Shunt (Fistula or Graft)	198	*58.4*
Length of time on HD		
5 years or less	252	*74.3*
Over 5 years	87	*25.7*
On waiting list for transplant		
No	289	*85.3*
Yes	50	*14.7*
Unit		
Hospital based	127	*37.5*
Community based	212	*62.5*

Abbreviations: CVC, Central venous catheter; HD, hemodialysis

* Percentage differences due to missing data

Patients receiving dialysis in hospital-based units were, on average, older (mean age: 69.43 years, SD = 13.2) compared to those in community-based units (mean age: 64.03 years, SD = 13.9; p < .001). Additionally, a significantly higher proportion of patients in community-based units were either already on the transplant list or undergoing eligibility testing compared to those in hospital-based units.

### Interest in participating in dialysis treatment related care tasks

Overall, the majority of patients expressed interest in participating in simpler care tasks, such as measuring weight (n = 276, 81.4%) and blood pressure and pulse (n = 253, 74.6%). Additionally, approximately 30–40% indicated interest in taking part in more complex tasks, such as preparing the dialysis machine and equipment before treatment (Table 2).

**Table 2:** Patient interest in participating in care tasks.

Treatment task	Not interested		Maybe interested		Yes, interested		Already participate	
	N	%	N	*%*	N	%	N	*%*
Measuring weight	63	*18.6*	4	*1.2*	188	*55.5*	84	*24.8*
Measuring blood pressure and pulse	86	*25.4*	25	*7.4*	220	*64.9*	8	*2.4*
Preparing dialysis machine	206	*60.8*	24	*7.1*	98	*28.9*	11	*3.2*
Preparing equipment for connection	207	*61.1*	23	*6.8*	101	*29.8*	8	*2.4*
Programming machine	209	*61.7*	24	*7.1*	95	*28.0*	11	*3.2*
Cannulating/ Preparing CVC	241	*71.1*	25	*7.4*	62	*18.3*	11	*3.2*
Connecting and commencing treatment	219	*64.6*	16	*4.7*	93	*27.4*	11	*3.2*
Responding to machine alarms	196	*57.8*	26	*7.7*	103	*30.4*	14	*4.1*
Returning blood and disconnecting	230	*67.8*	16	*4.7*	82	*24.2*	11	*3.2*
Holding needle sites after removal/ closing up CVC	160	*47.2*	16	*4.7*	146	*43.1*	17	*5.0*
Injecting medications via machine	221	*65.2*	26	*7.7*	80	*23.6*	12	*3.5*

Abbreviations: CVC, Central venous catheter

Notably, 37 patients (10.9%) expressed no interest in participating in any care tasks, at any level (Table 4 and 6).

**Table 3:** Patient performance of care tasks over previous three dialysis treatments.

How often over the last 3 treatments have you performed the following care tasks?								
**Treatment task**	**Never**		**Sometimes**		**Usually**		**Always**	
	**N**	**%**	**N**	***%***	**N**	**%**	**N**	***%***
Measuring weight	227	*67.0*	1	*0.3*	3	*0.9*	108	*31.9*
Measuring blood pressure and pulse	315	*92.9*	9	*2.7*	3	*0.9*	12	*3.5*
Preparing dialysis machine	339	*100.0*						
Preparing equipment for connection	339	*100.0*						
Preparing access (washing shunt hand or switching CVC dressing)	204	*60.2*	9	*2.7*	3	*0.9*	123	*36.3*
Deciding ultrafiltration volume	204	*60.2*	27	*8.0*	15	*4.4*	93	*27.4*
Deciding duration of treatment	296	*87.3*	23	*6.8*	5	*1.5*	15	*4.4*
Programming machine	339	*100.0*						
Cannulating/ Preparing CVC	339	*100.0*						
Connecting and commencing treatment	339	*100.0*						
Responding to machine alarms	320	*94.4*	9	*2.7*	8	*2.4*	2	*0.6*
Returning blood and disconnecting	339	*100.0*						
Holding needle sites after removal/ closing up CVC	319	*93.8*	4	*1.2*	2	*0.6*	15	*4.4*
Injecting medications via machine	339	*100.0*						

Abbreviations: CVC, Central venous catheter

**Table 4:** Bi-variate analysis: interest in participating in care tasks.

	No interest in participating in any care task	*%*	Interest in participating in at least one care task	*%*	
	N 37	*10.90*	N 302	*89.1*	p-value^**^
Patient characteristics					
Demographics					
Age, years, mean (SD)	72.76	*13.81*	65.23	*13.69*	0.453
Education level					<0.001
Elementary school or less	23	*62.2*	78	*25.8*	
High school or above	14	*37.8*	224	*74.2*	
Sex					0.004
Male	12	*32.4*	173	*57.3*	
Female	25	*67.6*	129	*42.7*	
Religion					<0.001
Jewish	7	*18.9*	144	*47.7*	
Other	30	*81.1*	158	*52.3*	
Marital status					<0.001
Married/ in partnership	13	*35.1*	211	*69.9*	
Single/ Divorced/ Widowed	24	*64.9*	91	*30.1*	
Economic status					0.181
Average or below	26	*70.3*	241	*79.8*	
Above average	11	*29.7*	61	*20.2*	
Health literacy					0.002
Low	26	*70.3*	132	*43.7*	
Average and high	11	*29.7*	170	*56.3*	
Clinical characteristics					
Vascular access (CVC vs shunt)					0.103
CVC	20	*54.1*	121	*40.1*	
Shunt (Fistula or Graft)	17	*45.9*	181	*59.9*	
Length of time on HD					0.841
5 years or less	27	*73*	225	*74.5*	
Over 5 years	10	*27*	77	*25.5*	
Unit					0.084
Hospital based	9	*24.3*	117	*38.9*	
Community based	28	*75.7*	184	*61.1*	

Abbreviations: CVC, Central venous catheter, HD, Hemodialysis

** p-values derived from chi-square, Fisher-exact test or independent t-test according to data type

**Table 5:** Bi-variate analysis of participation in two or more care tasks.

	Non-participation	*%*	Participation of any degree	*%*	
	N 203	*59.90*	N 136	*40.10*	p-value^**^
Patient characteristics					
Demographics					
Age, years, mean (SD)	66.4	*14.84*	65.53	*12.35*	0.012
Education level					0.911
Elementary school or less	60	*29.60*	41	*30.10*	
High school	88	*43.30*	61	*44.90*	
Higher education	55	*27.10*	34	*25.00*	
Sex					0.348
Male	115	*56.70*	70	*51.50*	
Female	88	*43.40*	66	*48.50*	
Religion					0.01
Jewish	102	*50.20*	49	*36.00*	
Other	101	*49.80*	87	*64.00*	
Marital status					0.229
Married/ in partnership	129	*63.50*	95	*69.90*	
Single/ Divorced/Widowed	74	*36.50*	41	*30.10*	
Economic status					0.047
Below average	27	*13.30*	15	*11.00*	
Average	142	*70.00*	83	*61.00*	
Above average	34	*16.70*	38	*27.90*	
Health literacy					0.152
Low	103	*50.70*	55	*40.40*	
Average	19	*9.40*	18	*13.20*	
High	81	*39.90*	63	*46.30*	
Clinical characteristics					
Vascular access (CVC vs shunt)					<0.001
CVC	107	*52.70*	34	*25.00*	
Shunt (Fistula or Graft)	96	*47.30*	102	*75.00*	
Length of time on hemodialysis					0.002
5 years or less	163	*80.30*	89	*65.40*	
Over 5 years	40	*19.70*	47	*34.60*	
Unit					0.107
Hospital based	69	*34.00*	58	*42.60*	
Community based	134	*66.00*	78	*57.80*	
Interest in participating					0.002
No interest	31	15.3	6	4.4	
Any level of interest	172	84.7	130	95.6	

Non-participation = participation in less than two care tasks

Abbreviations: CVC = Central Venous Catheter

** p-values derived from chi-square or independent t-test according to data type

**Table 6:** Patient characteristics associated with interest for participation in hemodialysis care tasks.

Variable	Odds Ratio	95% CI (lower)	95% CI (upper)	p-value
Sex	1.067	0.443	2.567	0.885
Education level (ref: low)				
high	0.36	0.146	0.897	0.028
Religion (ref: all others)				
Jews	2.375	0.890	6.338	0.084
Marital status (ref: in relationship)				
Single	0.268	0.121	0.596	0.001
Health literacy (ref: low)	1.403	0.492	3.316	0.476
High				
Vascular access (ref: CVC)				
Shunt	1.537	0.731	3.232	0.257
Hospital or community unit (ref: hospital)				
Community unit	0.720	0.297	1.746	0.467

Abbreviations: CVC  =  Central Venous Catheter

### Actual participation in dialysis treatment care tasks

Following the study’s definition of participation as engagement in at least two care tasks, regardless of the extent of involvement, our analysis showed that 136 patients (40.1%) patients met this criterion, participating in at least two care tasks to some degree. All of these care tasks were simple care tasks. A clear floor effect was observed, as seven of the care tasks – all classified as complex – were not performed by any of the patients (Table 3).

### Association of patient-related factors with interest and actual participation in care tasks

In analyzing the unadjusted relationships between demographic characteristics and interest in care task participation, we found that higher education levels, male gender, Jewish religion (compared to other religious affiliations), and being married or in a partnership (compared to being single) were associated with greater interest in participation. Additionally, individuals with average or high health literacy expressed greater interest than those with low health literacy.

The clinical factors of vascular access type, dialysis duration, and unit location (community vs. hospital) did not show statistically significant correlations with interest in care task participation (Table 4). These findings represent crude associations observed in our initial bivariate analysis.

Notably, while not statistically significant, a greater proportion of patients who were uninterested in participation were from community-based units (75.7%), compared to those who expressed at least some interest, of whom 61% were from community-based units.

In an unadjusted bivariate analysis examining the association between demographic characteristics and actual participation in at least two care tasks, several significant factors emerged. Specifically, younger age, non-Jewish religious affiliation, and higher economic status were statistically significantly associated with greater participation.

On the clinical side, patients with arteriovenous shunts and those who had been on dialysis for less than five years were also found to be significantly more likely to participate in care tasks (Table 5).

As presented in tables 4 and 5, no statistically significant differences were observed in interest or actual participation between patients in community-based and hospital-based units (Tables 4 and 5). To further investigate whether the relationship between interest and participation varied by unit type, we conducted a sub-analysis.

Our results show that while 93% of hospital-based patients expressed interest in participation and 46% actively participated, 87% of community-based patients expressed interest, yet only 36.5% participated. Given the relative participation rate among hospital-based patients, we would have expected a higher proportion of community-based patients to participate (42%).

### Patient characteristics associated with interest in participation in care tasks

To examine the adjusted associations between personal and organizational factors (sex, education level, religion, marital status, health literacy, vascular access, and unit type – hospital vs community-based), we conducted a logistic regression analysis. The model fit was satisfactory, as assessed using the Hosmer and Lemeshow test (*p* = .404).

As shown in Table 6, only education level and marital status were significantly associated with interest in participating in care tasks, while sex, religion, health literacy, vascular access and unit type were not. Specifically, higher education levels and being in a relationship were positively associated with interest, indicating an increased likelihood of participation among these individuals, controlling for all other variables in the model.

### Patient characteristics associated with participation in hemodialysis care tasks

The logistic regression analysis examining predictors of actual participation in simple hemodialysis care tasks included six independent variables: age, religion, economic status, vascular access, length of time on haemodialysis and unit location. The model demonstrated satisfactory fit, as assessed using the Hosmer and Lemeshow test (*p* = .134).

As shown in Table 7, all variables except age remained statistically significant when controlling for other factors. Religion emerged as a significant predictor, with individuals identifying as Jewish displaying a significantly lower likelihood of participating in treatment-related care tasks.

**Table 7:** Patient characteristics associated with participation in hemodialysis care tasks.

Variable	Odds Ratio	95% CI (lower)	95% CI (upper)	p-value
Age	1.004	0.984	1.025	0.663
Religion (ref: all others)				
Jews	0.293	0.0159	0.543	>0.001
Economy level (ref: low)				
High	2.671	1.446	4.933	0.002
Vascular access (ref: CVC)				
Shunt	3.313	1.979	5.547	>0.001
Length of time on hemodialysis (ref: < 5 years)				
>5 years	1.858	1.064	3.244	0.030
Unit location (ref: Hospital)				
Community unit	0.435	0.248	0.762	0.004
Interest in participating (ref: no interest)				
Interested	6.011	2.186	16.526	>0.001

Abbreviations: CVC = central venous catheter

Patients with higher economic status, those using arteriovenous shunts for dialysis access, and those with more years of hemodialysis treatment were more likely to participate in simple treatment tasks compared to individuals with lower economic status, those using central venous catheters (CVCs), and those with fewer years of hemodialysis experience.

Furthermore, individuals who expressed interest in participation were significantly more likely to engage in care tasks. Notably, patients dialyzing in community-based units were significantly less likely to participate in care tasks compared to those receiving treatment in hospital-based units.

## Discussion

This study provides new insights into the interest and actual participation of patients in hemodialysis care, as well as the sociodemographic, clinical, and organizational factors associated with participation. Our findings reveal that while most patients expressed interest in participating in treatment-related care tasks, only a minority actively participated over the previous few treatments. Higher education levels and being in a partnership were associated with greater interest in participation.

Interestingly, the characteristics of those who were most likely to express interest differed from those who were most likely to have actually participated. Actual participation was significantly associated with non-Jewish religious affiliation and higher economic status. Limited research exists on how cultural differences influence self-care behaviour [20]. Additionally, patients with arteriovenous access, more than five years on dialysis, and those treated in hospital-based units were more likely to have participated. These results are consistent with Fotheringham et al. (2021), who found that patients with higher education, higher health literacy levels, and longer dialysis duration self-performed five or more treatment-related tasks [13].

Our hypothesis that younger patients with higher health literacy would exhibit higher interest and participation levels was not supported. Interestingly, our unexpected results align with broader trends in the literature, which suggest that patients with more advanced disease tend to demonstrate greater self-care behaviors than those in earlier stages of chronic kidney disease [24,25]. This discrepancy highlights the need for targeted efforts to bridge the gap between patient interest and actual participation, emphasizing the importance of engaging patients who express interest but do not transition to active involvement.

Increasing patient participation in in-center care may support greater adoption of home dialysis, as seen in efforts like the ESRD Treatment Choices (ETC) Model, which aimed to boost home dialysis utilization [26]. Pre-dialysis nephrology care may also play an important role in enhancing patient participation; however, this study did not investigate how utilized or accessible pre-dialysis care is across different settings in Israel, or how various factors (e.g., patient awareness, healthcare provider referrals, and availability of services) may impact its use and, consequently, patient participation.

Beyond the individual characteristics, a significant difference was observed between hospital and community-based patients, with the latter expressing lower interest and participating less. These findings contradict our hypothesis, which was based on prior research suggesting that patients in community-based units tend to be younger and healthier than those in hospital-based units [21], and thus would be more likely to participate in treatment tasks. However, in our sample, patients in community-based units were less likely to participate, even when controlling for age, economic level and length of time on dialysis. The reasons for these disparities remain unclear but suggest that organizational factors, such as unit culture and protocols, play a role in participation beyond personal patient factors [20,26]. This paper contributes to the existing knowledge on patient participation by utilizing a comprehensive measure of participation, assessing 14 hemodialysis treatment-related care tasks that have been documented in the literature and reported by patients [13,16]. However, seven (50%) of these care tasks were not performed at all in our sample. The observed floor effect in patient participation measurements may be due to unit policies that restrict patient involvement, rather than a mismatch between patient’s preferences and actual experience [27]. In such cases, it is expected that a substantial proportion of patients would report no participation in specific tasks.

This study found that 57% of patients expressed interest in participating in hemodialysis-related treatment tasks, with a nearly threefold difference in interest between simple and complex tasks. To promote patient autonomy, staff should offer patients opportunities for self-care, based on their individual interest and capabilities [28].

To better understand the discrepancy between patients’ expressed interest and actual participation, further research is warranted. Future studies should employ qualitative or mixed methods approaches to examine factors such as patient and healthcare provider motivation, the organizational environment, and the impact of policies on patient motivation and engagement. Additionally, further studies are required to explore the cost implications – both financial and health related – of increasing in-center self-care. These studies should assess potential benefits, such as cost savings and improvements in patient outcomes, alongside the challenges of implementation. These findings could inform future policies for improving self-care practices in hemodialysis settings.

## Limitations

This study has several limitations that should be noted. The generalizability of the findings is limited due to self-selection bias, as participation was voluntary, and the study was conducted in only four hemodialysis units in Israel. Furthermore, as this study employed a cross-sectional design, it can only identify associations between variables and cannot establish causal relationships. Additionally, this study relied on patient-reported data, limiting the ability to assess clinical complexity. The health literacy measure used may not have fully captured the specific literacy required for patient participation in hemodialysis treatment, which could explain the lack of significant findings in this area, due to its lack of contextual specificity to dialysis-related tasks. Future research should examine the extent to which clinical and functional status influences participation in hemodialysis care tasks and explore more targeted measures of dialysis-specific health literacy.

## Conclusions

Most in-center dialysis patients do not actively participate in hemodialysis treatment-related care tasks, despite expressing interest -- particularly in simple tasks that align with routine chronic self-care. When designing interventions to enhance patient participation, it is important to consider factors such as patient interest, demographic characteristics, and the organizational context in which care is provided.

Patient participation may be more effectively encouraged among individuals who are relatively new to hemodialysis (i.e., those who have recently initiated but are already established in treatment) and those with arteriovenous shunts rather than catheters. The underlying reasons for this pattern—whether related to patients’ health status or the relative ease of self-care with shunt-based access—remain unclear.

Although younger, healthier patients may seem like logical candidates for participation-focused interventions, our findings challenge this assumption. Instead, our results suggest that organizational and cultural factors have a substantial impact on both interest and actual participation. Increasing patient participation in hemodialysis treatment-related tasks could lead to better alignment between care models and patient preferences, ultimately improving patient-centered outcomes.
